# Corona Tracing Apps – Eine Analyse und Strukturierung des europäischen Marktes

**DOI:** 10.1365/s40702-021-00753-9

**Published:** 2021-06-28

**Authors:** Alfred Benedikt Brendel, Maike Greve, Kristin Masuch, Simon Trang

**Affiliations:** 1 Technische Universität Dresden, Dresden, Sachsen Deutschland; 2 Universität Göttingen, Göttingen, Niedersachsen Deutschland

**Keywords:** COVID-19, Tracing Apps, Kontaktverfolgung, Funktionalität, Morphologische Analyse, COVID-19, Tracing Apps, Contact Tracing, Functionality, Morphologic Analysis

## Abstract

Apps für die Kontaktnachverfolgungen – so genannte Corona Tracing Apps – stellen einen essentiellen Teil der nationalen Strategien zur Bekämpfung des COVID-19 Virus dar. Mithilfe dieser Technologie sollen Infektionsketten effektiver nachvollzogen und schnellstmöglich unterbrochen werden. Corona Tracing Apps lassen sich aus Perspektive der Technik, der Entwicklung und der Funktionalität auf verschiedenste Weise gestalten. Aufgrund der Vielfalt an Möglichkeiten wurden seit dem Beginn der Coronapandemie mehr als 40 verschiede Apps entwickelt und in Europa veröffentlicht. Diese Vielfalt an Technologie wird zum Problem, da die Effektivität von Corona Tracing Apps davon abhängt, wieviel Bürger*innen dieselbe App nutzen. Dieser Beitrag widmet sich der Vielfalt verschiedener App Konfigurationen. Auf Basis einer morphologischen Analyse untersuchen wir, in welchen Aspekten sich die Apps unterscheiden und zeigen anschließend, dass sich diese Apps in zwei Archetypen unterscheiden lassen.

## Abstract (en)

So called corona tracing apps are an essential part of national strategies to fight the coronavirus. Contact tracing apps aim to trace infection chains and interrupt them as quickly as possible more effectively. Corona tracing apps can be designed in a variety of ways from a technological, a development, and a functional perspective. Due to the diversity of options, more than 40 different apps have been developed and released in Europe since the beginning of the corona pandemic. This diversity of technology becomes a problem because the effectiveness of corona tracing apps depends on how many citizens use the same app. This paper addresses the diversity of different app configurations. Based on a morphological analysis, we investigate in which aspects the apps differ and then show that these apps can be distinguished into two archetypes.

## Einleitung

Um die Verbreitung des COVID-19 Virus zu unterbinden, gilt es die Infektionsdynamik zu unterbrechen. Ein zentrales Instrument ist dabei die Kontaktverfolgung infizierter Personen (Robert Koch-Institut 2020) mit Hilfe sogenannter mobiler COVID-19 Contact Tracing (CCT) Applikationen (App). CCT Apps unterstützen bei der Kontaktverfolgung, indem sie automatisiert die Kontakte der Nutzer*innen erfassen. Wurden Nutzer*innen positiv auf das COVID-19-Virus getestet, können die erfassten Kontakte über das Infektionsrisiko informiert werden und sich in Selbstquarantäne begeben bzw. testen lassen (Ferretti *et al.* 2020).

Seitdem die COVID-19 Infektionszahlen in Europa im März 2020 drastisch anstiegen und die Regierungen erste Maßnahmen (bspw. Lockdown) anordneten, wurden verschiedene CCT Apps entwickelt und veröffentlicht. Daneben haben sich unterschiedliche Implementierungsansätze und Tracing Protokolle etabliert, auf deren Basis die CCT Apps aufbauen. Zum Beispiel spielt das in Partnerschaft von Google und Apple entwickelte „Exposure Notification Framework API“ eine zentrale Rolle, da diese von vielen der CCT Apps verwendet wird und eine essenzielle Komponente darstellt. Neben dem Einsatz dieses Framework gibt es diverse andere Optionen und Möglichkeiten eine CCT App zu gestalten und zu vertreiben. So kann eine CCT App per Bluetooth und/oder die GPS-Koordinate versuchen einen Kontakt mit einem Infizierten Menschen zu erkennen. Außerdem kann eine solche App staatlich oder privat-wirtschaftlich entwickelt und angeboten werden.

Infolge der Vielzahl an Gestaltungsmöglichkeiten und der rasanten Zunahme der verfügbaren CCT Apps steigt die Komplexität für Entwickler*innen hinsichtlich der Technik, Funktionen und Entwicklung. Gleichwohl zeigt es die Notwendigkeit einer strukturierten Übersicht, um Archetypen in der App-Gestaltung zu identifizieren. Basierend auf diesen Archetypen könnte anschließend erforscht werden, für welche Anwendungsgebiete sich bestimmte App-Gestaltungen anbieten oder wie Nutzer*innen auf bestimmte Konfigurationen reagieren. Vor diesem Hintergrund ist es das Ziel dieses Artikels, die Gestaltungsmerkmale von aktuellen europäischen CCT Apps systematisch abzuleiten und Archetypen zu identifizieren. Mit diesem Forschungsvorhaben werden folgende Forschungsfragen adressiert:

### FF1:

Anhand welcher Merkmale lassen sich europäische CCT Apps hinsichtlich ihrer Technik, Entwicklung und Funktionalität differenzieren?

### FF2:

Welche CCT App Archetypen lassen sich identifizieren?

Zur Beantwortung dieser Forschungsfragen wurde für diesen Artikel eine systematische Suche nach europäischen CCT Apps durchgeführt und anschließend die gesammelten 43 CCT Apps einer morphologischen Analyse unterzogen. Basierend auf den entwickelten Dimensionen und Eigenschaften wurde anschließend eine Clusteranalyse durchgeführt, wobei zwei Archetypen identifiziert werden konnten.

Im nachfolgenden Kapitel wird ein kurzer Überblick über aktuelle Forschung rund um CCT Apps gegeben. Anschließend wird die methodische Vorgehensweise, mittels welcher die relevanten CCT Apps identifiziert, analysiert und ausgewertet werden, beschrieben. In Kap. 4 werden die Dimensionen und Eigenschaften des entwickelten morphologischen Kastens präsentiert. Ergänzend werden in Kap. 5 die Ergebnisse der Clusteranalyse dargestellt. Abschließend werden die Ergebnisse in Kap. 6 diskutiert. Kap. 7 schließt diesen Artikel mit einem kurzen Fazit.

## Aktuelle Forschung und Anwendung von Tracing Apps

Von Beginn der COVID-19 Pandemie wurde auf bewährte Verfahren des Nachverfolgens von Kontaktpersonen zurückgegriffen. Diese wurden bereits bei anderen Infektionskrankheiten, wie beispielsweise Tuberkulose, angewendet (Kiehl 2015). Während der COVID-19 Pandemie wird der digitalen Nachverfolgung eine zentrale Bedeutung zugesprochen, welche auch wesentliche Neuerungen im CCT mit sich bringt. Insbesondere die sogenannten Tracing Apps, welche eine digitale Nachverfolgung durch mobile Applikationen ermöglichen und vordergründig die Gesundheitsämter bei ihrer Arbeit unterstützen sollen, sind eine Innovation der COVID-19 Pandemie (Robert Koch-Institut 2020). Ihre zentrale Rolle bei der Nachverfolgung der Infektionsketten begründet sich dadurch, dass sich das Virus unter der Annahme, dass kein vollständiger Lockdown angeordnet wird oder alle gefährdeten Personen geimpft sind derart schnell verbreitet, als dass es mittels einer manuellen Kontaktverfolgung lückenlos nachverfolgt werden könnte. Dieser Effekt wird auch von Ferretti *et al.* (2020) in ihren Modellrechnungen bestätigt und es wird dargelegt, dass die Pandemie mittels der digitalen CCT Apps nachverfolgt und kontrolliert werden könnte. Sie zeigen, dass die Nutzung von Tracing Apps einen positiven Effekt bei der Eindämmung der COVID-19-Pandemie haben können. Die Effektivität von CCT Apps zur Kontaktverfolgung hängt allerdings überproportional von der Anzahl der tatsächlichen Nutzer*innen ab. Somit wird die breite Akzeptanz von CCT Apps in der Bevölkerung zu einer funktionalen Voraussetzung (Trang et al. 2020). Durch spezifische Eigenschaften von CCT Apps lassen sich hierbei drei zentrale Herausforderungen für eine funktionierende digitale Nachverfolgung ableiten.

Die erste Herausforderung besteht darin, dass das Motiv zur Nutzung von Tracing Apps ein anderes ist als das von anderen Apps im Gesundheitsbereich. Fitness- und Ernährungsapps konzentrieren sich bspw. Darauf, die Gesundheit der Nutzer*innen zu verbessern, indem sie es ermöglichen individuelle Werte zu ermitteln und zu optimieren. Tracing Apps hingegen dienen nicht nur dem Nutzer*innen selbst, sondern insbesondere auch der Gesellschaft. Somit ermöglichen Tracing-Apps Nutzer*innen zwar informiert über potenzielle Hochrisikokontakte zu sein, jedoch bietet die Anwendung keine Möglichkeit riskante Kontakte zu vermeiden. Vielmehr steht der gesellschaftliche Faktor dem individuellen gegenüber, in dem eine Tracing App dazu beiträgt, eine breite Abdeckung der Kontaktverfolgung zu ermöglichen und andere darüber zu informieren, ob sie möglicherweise infiziert sind (Trang et al. 2020).

Die zweite Herausforderung besteht darin, dass die Tracing Apps für ihre Funktionalität Zugriff auf die persönlichen Daten der Nutzer*innen haben müssen. Hierbei gibt es bei den Tracing Apps zwei zentrale Möglichkeiten: Erhebung von hoch sensiblen Daten oder Erhebung von weniger sensible Daten. Somit unterscheiden sich die Tracing-Apps darin, dass einerseits Infektionsketten und somit die Kontakte verfolgt werden, in dem das GPS eines Smartphones verwendet wird. Dadurch werden sensible, standortbasierte Daten erhoben. Alternativ kommt die Bluetooth Technologie zum Einsatz, um Benutzer*innen in der Nähe zu identifizieren und zu speichern und später rückwirkend zu kontaktieren. Auf diese Weise ist der tatsächliche Standort nicht bekannt, weswegen auf diesem Wege keine sensiblen standortbezogenen Daten der einzelnen Personen erfasst werden müssen. Zusätzlich unterscheidet sich auch die Speicherung der erhobenen Daten. Entweder erfolgt eine Speicherung der Kontaktdaten mit einem hohen Maß an Kontrolle, in dem die Daten vollständig und ausschließlich auf dem Smartphone gespeichert werden oder es erfolgt eine Speicherung ohne Benutzerkontrolle auf einem zentralen Server (Trang et al. 2020).

Die dritte Herausforderung besteht darin, dass die Tracing-App dauerhaft aktiviert sein müssen, damit Daten in Echtzeit abgerufen werden können. In diesem Punkt unterscheiden sich die Apps ebenfalls darin, wie viel der Nutzende zur kontinuierlichen Aktivität beitragen muss. Hierzu zählen bspw., ob eine aktive Benutzerinteraktion erforderlich ist, um die App aktiv zu halten oder sogar regelmäßige Updates durchgeführt werden müssen, um die Grundfunktionalität der App zu gewährleisten (Trang et al. 2020).

Wie bereits einleitend betont kann zum momentanen Zeitpunkt eine große Heterogenität zwischen den Tracing-Apps verzeichnet werden. Jede Anwendung adressiert die erwähnten Herausforderungen auf eine eigene Weise und Entwickler*innen sind bemüht, die jeweilige Tracing-App für die Allgemeinheit zu optimieren.

## Methodisches Vorgehen

In den folgenden Unterkapiteln wird das methodische Vorgehen beschrieben. Als Erstes wird beschrieben, wie die CCT Apps identifiziert wurden, bevor auf die morphologische Analyse der Eigenschaften der Apps eingegangen wird.

### Ermittlung von Contact Tracing Apps

Um eine CCT App Sammlung aufzubauen, wurde eine strukturierte Suche durchgeführt. Als Quellen zur Identifikation der App wurden öffentliche Datenbanken verwendet: die Datenbank der European Commission (2020) und Council of Europe (2020). Duplikate wurden entfernt und um der Aktualität des Themas gerecht zu werden, wurde zusätzlich eine Onlinerecherche via der Google-Suchmaschine durchgeführt. Die Untersuchung wurde im Dezember 2020 durchgeführt. Dies führte zu einem endgültigen Datensatz mit 43 Corona Tracing Apps aus 31 verschiedenen europäischen Ländern:

*Coronalert (Beligien); ViruSafe (Bulgarien); Smittestopp (Dänemark); Corona-Warn-App, Ito, OHIOH (Deutschland) HOIA (Estland); Koronavilkku (Finnland); Alertanoo, StopCovid, TousAntiCovid (Frankreich); Exo (Griechenland); Covid Tracker (Irland); Rakning C‑19 (Island); CovidApp – Covid Community Alert, diAry – Digital Arianna; Immuni, SM-COVID-19, STOPcovid19 (Italien); Stop COVID-19 (Kroatien); Apturi Covid (Lettland); Korona Stop LT (Lettland); COVID Alert (Malta); Coronamelder, PrivateTracer (Niederlande); StopCOVID (Nord Irland); StopKorona! (Nord Mazedonien); Smittestopp (Norwegen); Stopp Corona (Österreich); STOP COVID – ProteGO Safe (Polen); STAYAWAY COVID (Portugal); Gosuslugi. Covid Tracker, Social Monitoring (Russland); SwissCovid (Schweiz); OstaniZdrav (Slovenien) COVID-19.eus, Radar COVID (Spanien); eRouska 2.0 (Tschechien); Hayat Eve Sığar (Türkei); VirusRadar (Ungarn); NHS COVID-19; Protect Scotland (Vereinigtes Königreich); CovTracer (Zypern).*

Zur weiteren Analyse der ermittelten Apps wurde eine individuelle Recherche für jede App durchgeführt. Zuerst wurden die zu den Apps gehörigen Webseiten auf relevante Informationen untersucht. Besonders integrierte FAQs, die Allgemeinen Geschäftsbedingungen sowie die Datenschutzrichtlinien boten Informationen zur Verwendung und Verarbeitung der Daten. Des Weiteren wurde der Quellcode (sofern öffentlich verfügbar) eingesehen und mit den bisherigen Informationen abgeglichen.

### Morphologische Analyse

Zur Analyse der gesammelten CCT-Apps wurde eine morphologische Analyse durchgeführt. Die morphologische Analyse ist eine Technik, die den Grundgedanken verfolgt, ein komplexes Problem in einzelne unabhängige Dimensionen zu zerlegen, um es in eine überschaubare Struktur zu bringen und anschließend Lösungen zu präsentieren, die aus kombinierten charakteristischen unabhängigen Eigenschaften der zuvor getrennten Dimensionen bestehen (Ritchey 2011). Wir wenden die Methode für den Entwurf des morphologischen Kastens an und folgen dabei dem Vorgehensmodell nach Ritchey (2011) mit den folgenden fünf Schritten: (1) Identifizierung der relevanten Dimensionen, (2) Ermittlung der Eigenschaften der jeweiligen Dimensionen, (3) Entwerfen eines morphologischen Kastens, um alle charakteristischen Eigenschaften pro Dimension zu spezifizieren, (4) Analysieren der resultierenden Kombinationen von charakteristischen Eigenschaften (durch Clusteranalyse) und (5) Verwenden des Modells. Die grundlegende Notwendigkeit einer solchen Analyse liegt in der komplexen und undurchsichtigen Natur der CCT Apps und der Vielfalt am europäischen Markt. Wir konzentrieren uns auf die Identifikation der Dimensionen und deren Charakteristika.

## Analyse der Corona Tracings Apps

**Abb. 1:**
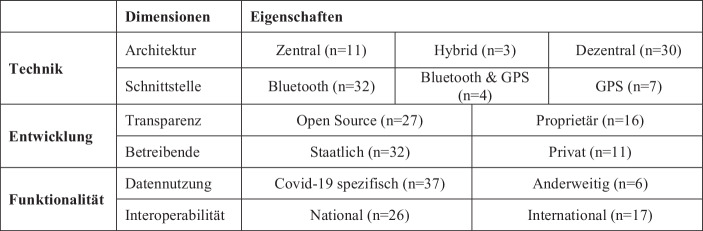
Morphologischer Kasten von Gestaltungs- und Vertriebsmöglichkeiten von CCT Apps (*n* *=* *43)*

CCT-Apps lassen sich anhand von sechs Dimensionen charakterisieren (siehe Abb. 1). Hierzu zählen zum einen technische Eigenschaften zur Architektur, der Nutzung von Schnittstellen und der Frage nach der Transparenz des Quellcodes. Zum anderen ist zu unterscheiden, wer der Herausgeber der App ist, ob neben der Kernfunktionalität der Kontaktverfolgung auch weitere Funktionalitäten integriert sind und ob die App einen nationalen oder internationalen Funktionsumfang hat.

### Technik

Für die Erfassung von Kontakten existieren verschiedene technische Ansätze, die entweder auf die GPS-Schnittstelle, die Bluetooth-Schnittstelle oder eine Kombination beider Schnittstellen setzen (Legendre et al. 2020). Bei der positionsbasierten Erfassung greift die App auf die GPS-Schnittstelle des Mobiltelefons zu. Die App zeichnet geographische Positionen der Nutzer*innen auf und erstellt ein Bewegungsprofil. Sobald sich ein Nutzender mit dem Coronavirus infiziert hat und dies der App mitteilt, wird für einen vorgegebenen Zeitraum abgeglichen, welche anderen Nutzer*innen sich zur selben Zeit am selben Ort befunden haben. Die näherungsbasierte Kontaktnachverfolgung benötigt keine Positionsdaten der Nutzer*innen. Über die Bluetooth-Schnittstelle wird erfasst, welche anderen Bluetooth-fähigen Geräte in der Nähe sind. So werden die potenziellen Kontakte abgeleitet und in einer Liste gespeichert, welche beinhaltet, ob ein potenzieller Kontakt zwischen den Nutzer*innen bestanden hat. Hat sich eine Person infiziert, lassen sich über die Kontaktlisten die Kontakte nachverfolgen. Während der GPS-basierte Ansatz eine genauere Erfassung von Kontakten außerhalb von geschlossenen Räumen ermöglicht, kann der Bluetooth-basierte Ansatz auch in geschlossenen Räumen potenzielle Kontakte erkennen. In der Praxis existieren deshalb auch Apps, die beide Ansätze kombinieren.

Eine weitere zentrale Fragestellung bei der Ausgestaltung von CCT Apps ist die Architektur der Datenspeicherung und -verarbeitung (Ahmed et al. 2020). Hierbei lassen sich zentrale, dezentrale und hybride Architekturen identifizieren. Diese unterscheiden sich im Kern darin, welche Daten und Verarbeitungsschritt auf dem Mobiltelefon oder auf dem zentralen Server des App-Anbieters gespeichert und ausgeführt werden. Bei einer dezentralen Architektur findet die Speicherung der Kontakte und die Kontaktüberprüfung ausschließlich in der App statt. Der Server hat in diesem Modell lediglich die Aufgabe, die autorisierten Positivmeldungen an alle anderen Apps zu verteilen. Im zentralen Modell werden notwendige Berechnungen wie die Kontaktüberprüfung auf den Servern ausgeführt. Dazu müssen die Kontaktdaten an die Server übermittelt und dort abgespeichert werden. Bei hybriden Ansätzen wird eine Kombination aus zentraler und dezentraler Speicherung und Verarbeitung gewählt. Das Ziel dieser geteilten Ansätze ist es, einen Ausgleich zwischen dem Datenschutz und einer erhöhten Funktionalität der Kontaktnachverfolgung zu erreichen.

### Entwicklung

Transparenz beschreibt, ob der Quellcode der App veröffentlicht ist, oder ob dieser der Öffentlichkeit vorenthalten und damit proprietär ist. Jedoch steht Open Source hier nicht automatisch für das Lizenzmodell Open Source. Mitunter gibt es wenige Ausnahmen, bei denen der Quellcode zwar veröffentlicht wurde, lizenztechnisch trotzdem kein Open Source Modell darstellt und somit auch nicht beliebig genutzt und vervielfältig werden darf. Das Lizenzmodell wurde hierbei vernachlässigt und die Apps danach klassifiziert, ob die App als Open Source Projekt beschrieben wird.

Die Dimension Betreibende beschreibt, ob die CCT App durch eine staatliche Einrichtung veröffentlicht wurde. Häufig wurden die CCT Apps in Zusammenarbeit mehrerer Parteien entwickelt und veröffentlicht. Sobald eine dieser Parteien einer staatlichen Organisation zuzuschreiben ist, so wurde der Veröffentlichende als staatlich klassifiziert. Teilweise wurden die CCT Apps in direktem Auftrag einer Behörde entwickelt und veröffentlicht. Auch diese Fälle wurden entsprechend als staatlich klassifiziert. Ein Beispiel ist die österreichische App Stopp Corona, die durch das Österreichische Rote Kreuz mitentwickelt und veröffentlicht wurde, jedoch im Auftrag der Regierung. Darüber hinaus ließ sich die Klassifizierung der CCT App auch von der Tracing Spezifikation ableiten. Denn nur Gesundheitsämter und damit staatliche Institutionen sind in der Lage, die GAEN Framework API zu verwenden (Google 2020). Die meisten der identifizierten CCT Apps wurden von staatlichen Einrichtungen veröffentlicht.

### Funktionalität

Datennutzung beschreibt, ob die durch die App erhobenen Daten nur für Zwecke der COVID-19 Pandemie verarbeitet werden, oder ob eine anderweitige Nutzung der Daten möglich bzw. vorgesehen ist. Der Fokus liegt hier jedoch auf den persönlichen Daten, nicht auf metrischen Daten der App. Diese werden von fast allen Anbietern erhoben und nicht im Kontext von COVID-19 verarbeitet, sondern zum Tracken in der App und des Nutzerverhaltens. Diese zwei Kategorien an Daten, persönliche Daten und App Metriken, werden auch in den jeweiligen Datenschutzrichtlinien aufgeführt.

Die Dimension Interoperabilität beschreibt, ob die CCT App national oder international verfügbar ist. International beinhaltet dabei, dass diese App nicht nur beschränkt auf das Land des Herausgebers verfügbar ist, sondern auch über die Landesgrenzen hinweg. Eine Vielzahl der CCT Apps sind ausschließlich national verfügbar. Der Grund für die beschränkte Verfügbarkeit der CCT Apps auf nationaler Ebene liegt darin begründet, dass die Gesundheitsbehörden die Positivmeldung autorisieren bzw. verifizieren müssen und in Kommunikation mit der App stehen. Einige CCT Apps sind daher in ihrer Verfügbarkeit sowie Verwendung eingeschränkt.

## Synthese

Nachdem wir ein Verständnis für die unterschiedlichen Eigenschaften der europäischen CCT Apps erarbeitet hatten, analysierten wir auf empirischer Basis diese im Hinblick auf häufige Kombinationen der Eigenschaften. Mithilfe der Durchführung einer Clusteranalyse zielten wir darauf ab, Archetypen (Cluster) von Ausprägungen auf Basis von Ähnlichkeitsmaßen zu identifizieren. Apps innerhalb eines Archetyps sollen dabei möglichst ähnlich und möglichst unähnlich zu Apps der anderen Archetypen sein.

Die Entscheidung, wie viele Cluster verwendet werden sollen, ist eine der größten Herausforderungen dieser Analyse. In der Literatur wird häufig ein zweistufiges Verfahren empfohlen. Entsprechend wurde zunächst die Ward-Methode zur Identifizierung der Anzahl von Clustern verwendet, ein hierarchisches und statistisches Verfahren. Die definierten Dimensionen mit ihren Eigenschaften dienten als binäre Variablen zur Ähnlichkeitsberechnung zwischen zwei Apps unter Verwendung der quadrierten euklidischen Distanz, die für binäre Daten geeignet ist. Durch die Auswertung der deskriptiven Daten, die Verwendung eines Dendrogramms und die Durchführung der Ellbogenregel (basierend auf dem Screenplot) haben wir die entsprechende Anzahl von Clustern ermittelt. Unsere Ergebnisse identifizierten eine ideale Anzahl von zwei Archetypen. Anschließend wendeten wir die k‑means-Methode an. Dies ist ein iteratives Partitionierungsverfahren, welches mehrere Optimierungsrunden für eine bereits definierte Anzahl von Clustern durchläuft, bis die Summen der quadrierten Distanzen zum Mittelwertsvektor jeder Gruppe minimiert sind.

**Abb. 2:**
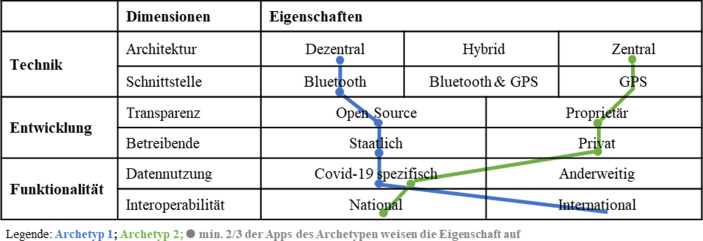
Morphologischer Kasten mit Archetypen

Abb. 2 zeigt den Morphologischen Kasten, in dem die Eigenschaften der Charakteristiken der beiden Archetypen durch einen „Faden“ verbunden sind. Der morphologische Faden verläuft durch die Eigenschaft, welche am häufigsten in den Apps des Archetyps vorzufinden ist. Die kreisförmigen Markierungen weisen darauf hin, wenn von einem Archetyp mehr als zweidrittel der Apps (eindeutige Mehrheit) die Eigenschaft aufweisen.

**Abb. 3:**
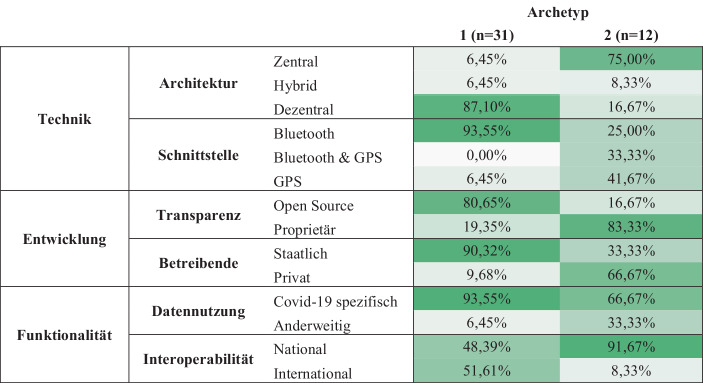
Verteilung der Apps in den einzelnen Archetypen

Abb. 3 zeigt einen detaillierten Einblick in die Verteilung der Charakteristika der jeweiligen Archetypen. Die prozentualen Angaben beziehen sich dabei auf den Anteil der App in den jeweiligen Archetypen, der diese Eigenschaft erfüllt. Je grüner die Einfärbung der jeweiligen Zelle, desto höher ist der Anteil der Apps mit dieser Eigenschaft in den jeweiligen Archetypen.

### Archetyp 1: CCT Apps zur Kontakterfassung

Unsere Auswertung zeigt, dass 31 Apps dem Archetyp 1 zugeordnet werden können. Somit legt sich dar, dass die Kombination aus Eigenschaften dieses Archetyps aktuell auf dem europäischen Markt überwiegen. Zu den dominanten Charakteristika der Apps gehören die dezentrale Architektur und die Kontakterfassung über Bluetooth. Die Entwicklung der Apps ist dabei staatlich veranlasst und der Code wird Open Source öffentlich zur Verfügung gestellt, um maximale Transparenz zu schaffen. Die Datennutzung ist ausschließlich Covid-19 spezifisch. Die Interoperabilität ist dabei teils international (51 %), aber auch national (49 %), sodass sich hier keine eindeutige Mehrheit zeigt.

Die Eigenschaften dieses Archetyps ermöglichen im Falle einer Infektion rückwirkend den Kontakt zu den App Nutzer*innen aufzunehmen, welche sich im näheren Radius der App in einer vergangenen Zeitperiode befunden haben. Es ist nicht möglich, die örtliche Lokalisation festzustellen oder diese Kontakte als Personen zu identifizieren. Ein Beispiel für eine solche App dieses Archetyps ist die deutsche Corona-Warn App.

Die deutsche Bundesregierung (2020) hat als offizielle Tracing App die „Corona-Warn-App“ am 16. Juni 2020 veröffentlicht. Diese wurde von SAP und der Telekom im Auftrag der Bundesregierung entwickelt. Die „Corona-Warn-App“ basiert auf dem dezentralen Contact Tracing und sowohl der Quellcode der App als auch der Backend-Infrastruktur werden vollständig als Open Source zugänglich gemacht. Die Corona-Warn-App wurde auf der Basis des Exposure Notification Framework („ENF“) von Apple und Google entwickelt, das Bluetooth Low Energy Technologie („BLE“) verwendet. Die App sammelt pseudonymisierte Daten von Mobiltelefonen in der Nähe über BLE. Sobald sich zwei Nutzende auf zwei Meter nähern und für fünfzehn Minuten oder länger in dieser Entfernung bleiben, tauschen ihre Apps anonymisierte IDs aus. Wenn Jemand positiv auf COVID-19 getestet wird, kann das Testergebnis in der App eingefügt werden. Die App informiert dann anonymisiert alle gespeicherten Kontakte. Die Daten werden lokal auf jedem Gerät gespeichert, wodurch der Zugriff und die Kontrolle über die Daten durch Behörden oder eine dritte Partei ausgeschlossen ist. Die EU arbeitet an der Interoperabilität der verschiedenen Tracing Apps. Aktuell kann die Corona-Warn-App in allen nationalen App-Stores der Europäischen Union sowie in denen der Schweiz, Norwegens, Großbritanniens und der Türkei heruntergeladen werden (Tab. 1).

**Tab. 1:** Zuordnung der Charakteristika der deutschen Corona-Warn App

Technik		Entwicklung		Funktionalität	
Architektur	Schnittstelle	Transparenz	Betreibende	Datennutzung	Interoperabilität
*Dezentral*	*Bluetooth*	*Open-Source*	*Staatlich*	*COVID-19 spezifisch*	*International*

### Archetyp 2: CCT Apps zur Kontaktverfolgung

Im Gegensatz zu Archetyp 1 zeigt der Archetyp 2 ein diverseres Bild an Eigenschaften. Insgesamt lassen sich 12 Apps diesem Archetyp zuordnen. Die dominanten Eigenschaften des Archetyps sind eine zentrale Architektur, während es kein klares Muster bezüglich der verwendeten Schnittstelle gibt. Im Vergleich zu Archetyp 1 lassen sich Apps, die GPS oder Bluetooth verwenden, mehrheitlich dem Archetyp 2 zuordnen. Die Mehrheit der Apps wurde von privaten Anbietern entwickelt (67 %) und stellt den Quellcode nicht öffentlich zur Verfügung (83 %). Es zeigt sich, dass sich die meisten Apps des Archetyps 2 nur national durchgesetzt haben und teilweise die Daten auch anderweitig verwendet werden (33 %).

Durch die hauptsächliche Verwendung von GPS und die hauptsächliche zentrale Architektur ist es prinzipiell möglich Bewegungsmuster abzuleiten. Somit könnte das Infektionsgeschehen lokalisiert werden. Im Falle einer Covid-19-Erkrankung ist es möglich, die Kontakte auf Basis von Standortinformationen rückwirkend zu benachrichtigen. Im Vergleich zu den Apps des Archetypen 1 weist dieser Archetyp eine starke Diversität auf. Da es nicht die *eine* typischen Konfiguration gibt, beschreiben wir zwei Apps im Detail.

Im Oktober 2020 wurde in Frankreich die bisherige CCT App aufgrund von Datenschutzbedenken überarbeitet und die neue Version unter dem Namen „TousAntiCovid“ veröffentlicht. Die Kontaktverfolgung funktioniert über Bluetooth. Im Falle einer Covid-19-Erkrankung sendet die App die Historie der angetroffenen Krypto-Identifikatoren an einen zentralen Server, ohne die eigenen Krypto-Identifikatoren offenzulegen. Jedes Smartphone, das die App heruntergeladen hat, überprüft regelmäßig mit diesem zentralen Server, ob seine Krypto-Identifikatoren zu den gefährdeten gehören. Die zentralen Server werden von der französischen Regierung verwaltet (Norton Rose Fulbright 2020) (Tab. 2).

**Tab. 2:** Zuordnung der Charakteristika der französischen „TousAntiCovid“ App

Technik		Entwicklung		Funktionalität	
Architektur	Schnittstelle	Transparenz	Betreibende	Datennutzung	Interoperabilität
*Zentral*	*Bluetooth*	*Proprietär*	*Privat*	*COVID-19 spezifisch*	*National*

Auch die norwegische CCT App „Smittenstopp“ basiert auf einer zentralen Architektur. Durch die Nutzung werden GPS und Bluetooth-Daten an das norwegische Institut für öffentliche Gesundheit gesendet. Diese Daten werden sowohl verwendet, um Kontakte zu infizierten Personen zu identifizieren, als auch um anonymisierte Statistiken zu erstellen, damit Bewegungsmuster in der Bevölkerung analysieren werden können. Wegen starker Kritik, dass alle Daten über einen längeren Zeitraum zentral gespeichert werden sollten und dass Nutzende sich nicht dafür entscheiden konnten, Daten nur für Kontaktverfolgungszwecke zu teilen, wurde die App im Sommer 2020 vom Markt genommen. Anfang 2021 wurde eine überarbeitete Version veröffentlicht (Euronews 2020; Norton Rose Fulbright 2020) (Tab. 3).

**Tab. 3:** Zuordnung der Charakteristika der norwegischen Smittenstopp App

Technik		Entwicklung		Funktionalität	
Architektur	Schnittstelle	Transparenz	Betreibende	Datennutzung	Interoperabilität
*Zentral*	*Bluetooth & GPS*	*Proprietär*	*Staatlich*	*COVID-19 spezifisch*	*National*

## Diskussion

Der vorliegende Beitrag ist eine der ersten Arbeiten, die sich mit den realen Implementierungen der CCT Apps beschäftigt. Bisher wurden in der Praxis, der Wissenschaft und den Medien vor allem die möglichen einzelnen Gestaltungsmöglichkeiten (z. B. zentrale versus dezentrale Architektur) diskutiert. In dieser Arbeit wird eine andere Perspektive eingenommen. Es wird auf der Metaebene analysiert, wie die verschiedenen Gestaltungsmöglichkeiten und Vertriebsstrategien kombiniert wurden. Mittels einer Clusteranalyse konnten zwei dominante Archetypen identifiziert werden, welche gegensätzliche Konfigurationen betrachtet. Der erste Archetype (CCT Apps zur Kontakterfassung) zeichnet sich vor allem durch eindeutige Eigenschaften aus, welche eine dezentrale Bluetooth-basierte Technik, eine staatlich-finanzierten und transparenten Entwicklung auszeichnet. Im Gegensatz dazu basiert Archetype 2 (CCT Apps zur Kontaktverfolgung) vor allem auf der Diversität in anderen Ansätzen. Darunter zählt eine zentrale Architektur, die Erhebung von GPS-basierten Daten und einer privaten proprietären Entwicklung. Die CCT Apps haben sich bisher hauptsächlich national durchgesetzte und wie unsere beiden Beispiele zeigen, stoßen diese auf Datenschutzkritik. Basierend auf diesen beiden Archetypen lassen sich verschiedene Implikationen ableiten. Es scheint sich ein implizierter Konsensus entwickelt zu haben, wie ein CCT App gestaltet und vertrieben werden sollte. Die Möglichkeiten für die Gestaltung und den Vertrieb einer CCT App werden nichtdisjunkt voneinander betrachtet und implementiert, sondern einige Eigenschaften werden als zusammengehörig angesehen. Zum einen gibt es die natürlichen technischen Umstände, welche zu einer Verbundenheit von Eigenschaften führen, wie eine dezentrale Architektur und der Einsatz von Bluetooth als Schnittstelle. Jedoch werden auch andere Eigenschaften häufig kombiniert bzw. können zusammen beobachtet werden, wie die Bereitstellung des Quellcodes als Open Source und staatliche Betreibende der CCT App. Hier bietet sich für die Wissenschaft die Möglichkeit die Prominenz von Kombinationsmöglichkeiten zu hinterfragen und Vor- und Nachteile für eine Abweichung von den Archetypen systematisch zu eruieren.

Für die Praxis bieten die Archetypen ein Framework für die Diskussion von CCT App-Gestaltungen. Entwickler*innen und Betreiber*innen von CCT Apps können ihre Gestaltung der App vor dem Hintergrund der identifizierten Archetypen diskutieren und Abweichungen explizit durchdenken. Somit ergibt sich ein Referenzrahmen für die Entwicklung von CCT Apps, welcher den Umfang einer „Trial-and-Error“ Phase reduzieren kann. Das ist von besonderer Bedeutung, da eine CCT App auf einen starken Netzwerkeffekt angewiesen ist. Eine langsame Zunahme der Nutzer*innen nach der Veröffentlichung der CCT App kann zu einem unmittelbaren Scheitern führen. Daher ist es notwendig, dass CCT Apps von Anfang an eine ausgereifte Gestaltung und Vertriebsstrategie aufweisen.

Abschließend ist anzumerken, dass sich CCT Apps im Archetyp 1 scheinbar durchgesetzt haben und ein Trend zu erkennen ist. Das deutet darauf hin, dass die Eigenschaften des ersten Archetypens erfolgsversprechender sind als die des zweiten Archetypens. Im Moment ist die Datenlage noch relativ unklar, aber wir glauben, dass es im Laufe der nächsten Jahre eindeutigere und verlässliche Daten geben wird. Mittels dieser Daten kann untersucht werden, welche Eigenschaftskonfigurationen zu einer höheren Nutzerzahl führen. Dennoch halten wir unsere Beobachtung für aktuell relevant.

An dieser Stelle möchten wir jedoch darauf hinweisen, dass Tracing Apps ein sehr dynamisches Technologiefeld sind, welches sich in kurzer Zeit, ausgelöst durch die Pandemie, ad hoc entwickeln musste, und sich stetig weiterentwickelt. Daher bietet unsere Analyse nur eine Momentaufnahme. Außerdem möchten wir auf die Limitationen der Methodik hinweisen. Die morphologische Analyse ist in ihrer methodischen Vorgehensweise nie vorständig und interpretativ ist. Sie bietet immer eine Kategorisierung ohne Anspruch auf Vollständigkeit. Zuletzt möchten wir auch auf die Limitationen der Clusteranalyse hinweisen. Die generische Unterteilung in zwei Archetypen verallgemeinert die vorliegende Datengrundlage, um die Balance aus inhaltlicher Erklärungskraft und Komplexität zu bieten.

## Fazit und Ausblick

Dieser Forschungsbeitrag befasst sich mit den verschiedenen europäischen CCT Apps, die im Rahmen der COVID-19 Pandemie entwickelt und auf den Markt gebracht wurden. Es wird untersucht, wie die unterschiedlichen Eigenschaften der Apps ausgeprägt sind und dahingehend zwei Hauptarchetypen anhand der Kombinationen der Eigenschaften identifiziert und definiert. Die Ergebnisse der vorliegenden Forschungsarbeit liefern wertvolle Erkenntnisse über die Ausgestaltung von CCT Apps im Kontext von COVID-19. Es zeigt sich, dass sich die CCT Apps des europäischen Marktes in Archetyp 1 und Archetyp 2 kategorisieren lassen. Zudem lässt sich feststellen, dass die beiden Archetypen eine unterschiedliche Verbreitung auf dem Markt aufweisen und sich zukünftig zeigen wird, ob der zweite Archetyp an Apps vom Markt verschwindet oder als Insellösung bestehen bleibt.

Neben diesen neuartigen Erkenntnissen sind einige wichtige Einschränkungen zu nennen, welche aber auch Möglichkeiten für zukünftige Forschung bieten. Eine besondere Herausforderung besteht gerade bei diesem speziellen Thema darin, dass die Ergebnislage sich schnell weiterentwickelt und die gesammelten Daten immer aktualisiert werden müssen. Somit kann nicht sichergestellt werden, dass alle Apps, die im Rahmen der Analyse verwendet wurden, noch existieren und keine neuen Apps hinzugekommen sind. Darüber hinaus kann, obwohl die Entwicklung der zwei Archetypen für die CCT Apps von zwei unabhängigen Forschern durchgeführt wurde, nicht garantiert werden, dass es keine anderen wichtigen Aspekte gibt, die zu einer weiteren Kategorie gehören könnten, da die Kodierung auf den ausgewählten Aspekten basiert. An dieser Stelle könnte zukünftige Forschung ansetzen und die Analyse der Archetypen erweitern und verfeinern und weitere mögliche Ebenen der Kategorisierung finden. Des Weiteren wurde nicht untersucht, ob die Eigenschaften der Archetypen tatsächlich zu einer Adaption oder Ablehnung der Apps führen. Dieser Aspekt scheint sich lediglich abzuzeichnen. Zukünftige Forschung sollte daher untersuchen, ob und wie verschiedene Eigenschaften von CCT Apps zur Adaption oder Ablehnung dieser führen.
